# Corneal transplantation at Tenwek Hospital, Kenya, East Africa: Analysis of outcomes and associated patient socioeconomic characteristics

**DOI:** 10.1371/journal.pone.0187026

**Published:** 2017-10-27

**Authors:** Michael C. Chen, Allen R. Kunselman, Christy M. Stetter, Sadeer B. Hannush, Benjamin W. Roberts

**Affiliations:** 1 Penn State Eye Center, Penn State College of Medicine, Hershey, Pennsylvania, United States of America; 2 Tenwek Hospital, Bomet, Bomet County, Kenya; 3 Department of Public Health Sciences, Penn State College of Medicine, Hershey, Pennsylvania, United States of America; 4 Wills Eye Hospital, Philadelphia, Pennsylvania, United States of America; London School of Hygiene & Tropical Medicine, UNITED STATES

## Abstract

**Purpose:**

To review the graft survival rate, visual outcomes, and patient demographics of primary penetrating keratoplasty performed at Tenwek Hospital, a mission hospital in rural Kenya.

**Methods:**

A retrospective review was performed of the clinical records of patients who underwent primary penetrating keratoplasty for optical purposes from January 2012 to October 2014. The graft survival rates were constructed using the Kaplan-Meier method, and the effect of clinical and socioeconomic characteristics on time to graft failure were examined using Cox regression models.

**Results:**

118 patients met the inclusion criteria. The most common indication for surgery was keratoconus (66.1%), followed by corneal scar (22.0%). Despite all patients giving a verbal commitment to do so, 40 patients (33.9%) failed to make it to followup one year postoperatively. Graft survival at one year, inclusive of all indications, was 85.8%. Of the different indications, keratoconus had the highest one-year graft survival rate of 89.9%. Compared to the preoperative uncorrected visual acuity, 85.3% achieved an improvement at one year. Compared to patients who had completed college or university, the risk of developing graft failure was 4.7 times higher among patients with less education (P = 0.01).

**Conclusions:**

Corneal transplantation at Tenwek Hospital can be performed with a reasonable chance of success at one year, particularly in cases of keratoconus and in patients with higher educational backgrounds. Adherence to followup recommendations proves to be a challenge in this patient population. Additional studies of larger patient populations with longer follow up periods in similar settings may be helpful in informing appropriate patient selection and maximizing successful outcomes of corneal transplantation in low-resource settings.

## Introduction

Corneal disease is one of the leading causes of visual impairment and blindness worldwide, with the majority of these people residing in the developing world [1, 2]. In many cases, corneal transplantation is the only treatment for visual rehabilitation. Unfortunately, corneal transplantation is not obtainable by the majority of the population in need. Worldwide, there is an estimated 12.7 million people in need of corneal transplantation, with only one cornea available for every 70 people [3]. This supply-demand imbalance is further accentuated in many developing countries where corneal transplantation procedures are either rarely performed or nonexistent, due to factors including a deficiency in eye care infrastructure and trained eye care personnel, lack of eye bank facilities and difficult access to donor tissue, and challenges that arise from unique characteristics of the patient population [4].

Where corneal transplantation is performed in the developing world, the most common type of transplant performed is penetrating keratoplasty [3]. Given the limited availability of donor corneas worldwide and particularly in many developing countries, assessing the outcomes of penetrating keratoplasty in low-resource settings is necessary to determine the utility of this procedure as well as how to prioritize patient candidacy to maximize the chance of graft survival and good visual outcome.

To the best of our knowledge, only one other report has been published on the outcomes of penetrating keratoplasty performed in a sub-Saharan African setting [5]. In this study we examine the graft survival rates, visual outcomes, and patient demographics of primary penetrating keratoplasty performed at Tenwek Hospital located in Bomet County, Kenya.

### Background of Tenwek Hospital

Tenwek Hospital is a tertiary care mission hospital located in rural Kenya, 4 hours (240 kilometers) west of the capital city Nairobi. Corneal transplantation services were established in 2010 and have been continued from that time forward by either full-time or visiting staff American ophthalmologists trained in corneal transplantation. Throughout the study period, at least one of these ophthalmologists was on site to provide followup care. Donor cornea tissue was imported from eye banks in the United States due to the limited local supply of tissue available. Surgery costs were subsidized, and most patients were charged a nominal fee for surgery to cover basic hospital costs. Patients received extensive preoperative counseling regarding the importance of followup. All patients provided verbal commitment to followup at Tenwek Hospital through one year postoperatively, after which if they were stable they had the freedom to seek future followup care with another provider if they desired. An electronic health record was implemented in 2012, allowing access to patient records and enabling this study.

## Materials and methods

A retrospective analysis of clinical records was performed in October 2015 of patients who underwent primary penetrating keratoplasty for optical purposes between January 2012 and October 2014. Transplants performed for tectonic purposes and repeat transplants were excluded from analysis. If an eye underwent a regraft during the study period, the data were excluded from analysis from that point forward. A corneal graft was considered failed if it had undergone a regraft or, in the absence of regraft, if there was irreversible loss of clarity due to edema, scarring, or neovascularization. Due to correlation between two eyes of the same patient [6], if a patient obtained corneal transplantation in both eyes during the study period, only the first eye was included for statistical analysis. Lost to followup, or failure to followup one year postoperatively, was defined as permanently ceasing to appear for examination at Tenwek Hospital prior to one year postoperatively, or ceasing to appear for examination prior to one year postoperatively and reappearing after postoperative month 18. Poor followup was defined as failing to appear for followup within one month of the time frame as instructed by the clinician. At the time of chart review, all patients were called on their mobile phones to answer a questionnaire regarding additional demographic details, including education level, occupation level, place of residence, and time of one-way travel to the hospital. Occupational level was classified according to the International Standard Classification of Occupations (ISCO) [7]. Age was classified using a cutoff of 14 years [8].

The graft survival rates were constructed using the Kaplan-Meier method.[9] The effect of clinical and socioeconomic characteristics on time to graft failure were examined one at a time using Cox regression models, and effect sizes were reported as a hazard ratio and 95% confidence interval (CI). Subsequently, a multivariable Cox regression model was constructed using the stepwise variable selection method. All analyses were performed using SAS software, version 9.4 (SAS Institute Inc., Cary, North Carolina, USA) or R software (R Foundation for Statistical Computing, Vienna, Austria).

The design of this study was reviewed and approved through the Tenwek Hospital Institutional Review and Ethics Committee and the Penn State College of Medicine Institutional Review Board. The requirement for informed consent was waived. All data was fully anonymized before access by the researchers.

## Results

Over the study period, primary penetrating keratoplasty was performed on 126 eyes of 118 patients by 6 surgeons. Of these patients, 108 were successfully reached by phone at the time of chart review and participated in a questionnaire regarding additional demographic data. For the purposes of this report, for the eight patients who had the procedure performed on both eyes, only the first eye was included in all analyses.

Seventy-seven patients (65.3%) were male and 41 patients (34.7%) were female. The mean age, in years, at the time of surgery was 30.1 (SD = 20.7), the median age was 22, and the age range was 6 to 92. Sixteen patients (13.6%) were age 14 or younger.

The clinical and socioeconomic characteristics of the population are shown in Table 1. The most common preoperative diagnosis was keratoconus (66.1%), the most common type of procedure was penetrating keratoplasty alone (89.0%), and 43.0% of the population had completed college or university.

**Table 1:** Clinical and socioeconomic demographics.

	N (%)
**Preoperative Diagnosis**	
*Keratoconus*	78 (66.1%)
*Corneal scar*	26 (22.0%)
*Bullous keratopathy*	13 (11.0%)
*Chemical injury*	1 (0.9%)
**Procedure Type**	
*PKP alone*	105 (89.0%)
*PKP/ECCE/IOL implantation*	6 (5.1%)
*PKP/IOL exchange*	5 (4.2%)
*PKP/ICCE*	2 (1.7%)
**Education**^**a**^^**,**^ ^**b**^	
*Did not complete primary school*	20 (18.7%)
*Completed primary school*	22 (20.6%)
*Completed secondary school*	19 (17.8%)
*Completed college or university*	46 (43.0%)
**Occupation**^**c**^	
*ISCO level 1*	9 (8.3%)
*ISCO level 2*	11 (10.2%)
*ISCO level 3*	15 (13.9%)
*ISCO level 4*	5 (4.6%)
*Student*	55 (50.9%)
*Unemployed*	13 (12.0%)

PKP = penetrating keratoplasty; ECCE = extracapsular cataract extraction; IOL = intraocular lens; ICCE = intracapsular cataract extraction; ISCO = International Standard Classification of Occupations (1 is least skilled, 4 is most skilled).

^a^Education refers to the highest level of education achieved at time of surgery.

^b^Questionnaire response not available in 11 patients.

^c^Questionnaire response not available in 10 patients.

Eighteen patients (16.7%) reported Nairobi as their place of residence. The median number of hours of travel one way to the hospital, with 25^th^ and 75^th^ percentiles, was 4.0 (2.8, 6.0).

The median length of followup, with 25^th^ and 75^th^ percentiles, was 18 (10, 27) months, and the range of followup was 0–40 months. Forty patients (33.9%) failed to followup one year postoperatively. An additional 13 patients (11.0%) exhibited poor followup patterns.

**Fig 1:**
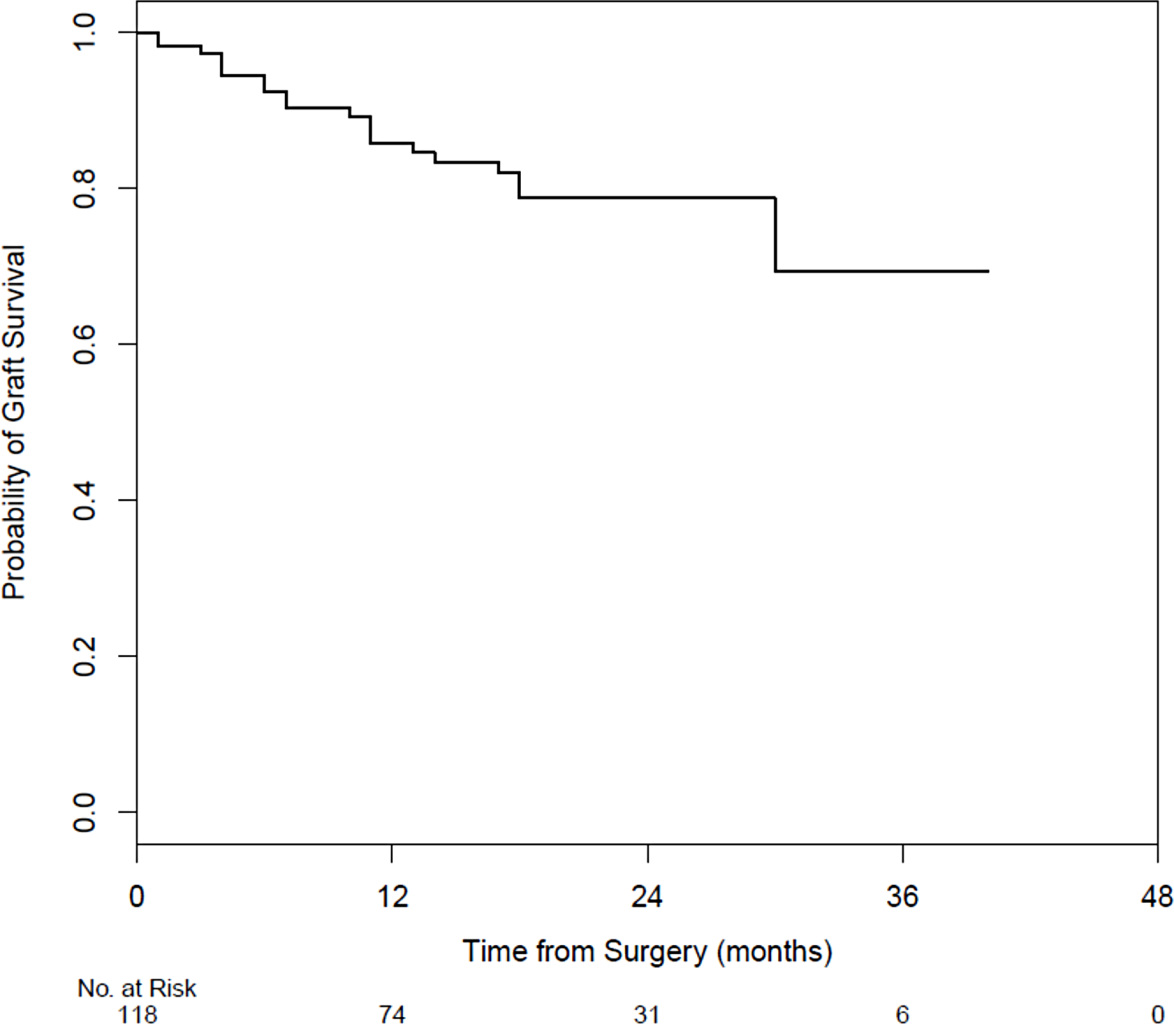
Kaplan-Meier graft survival.

The Kaplan-Meier graft survival is summarized in Fig 1. The one-year graft survival rate, inclusive of all preoperative diagnoses, was 85.8% (95% CI: 79.1% to 93.0%). One-year graft survival rates according to diagnosis and age category are shown in Table 2.

**Table 2:** 1-year graft survival by preoperative diagnosis and patient age.

	N Total	N Failed	N Censored	N at Risk	Rate (95% CI)
**Overall**	118	14	30	74	85.8% (79.1, 93.0)
**Diagnosis**					
Keratoconus	78	7	16	55	89.9% (83.0, 97.3)
Bullous keratopathy	13	1	8	4	85.7% (63.3, 100.0)
Corneal scar	26	5	6	15	76.6% (60.6, 97.0)
Chemical injury	1	1	0	0	0
**Age**					
≤ 14	16	3	7	6	72.9% (50.3, 100.0)
> 14	102	11	23	68	87.5% (80.8, 94.7)

CI = Confidence Interval

The effects of clinical and socioeconomic characteristics on graft failure, as determined by individual Cox regression models, are shown in Table 3.

**Table 3:** Association of clinical and socioeconomic characteristics with graft failure.

Characteristic	Level of characteristic	Hazard Ratio (95% CI)	P-value
**Preoperative diagnosis**	Keratoconus	1.0	
	Corneal scar	3.3 (1.3, 8.5)	0.01
	Bullous keratopathy	3.6 (1.0, 13.4)	0.06
	Chemical injury	25.1 (2.9, 218.3)	0.004
**Age (years)**	>14	1.0	
	≤14	1.5 (0.4, 5.1)	0.52
**Preoperative visual acuity in contralateral eye**	≥6/18	1.0	
	<6/18	1.1 (0.5, 2.6)	0.86
**Procedure type**	Penetrating keratoplasty alone	1.0	
	Penetrating keratoplasty combined	1.8 (0.4, 7.9)	0.43
**Hours from hospital**		1.0 (0.9, 1.2)	0.55
**Nairobi residence**	Yes	1.0	
	No	4.0 (0.5, 29.8)	0.18
**Education level**	Completed college or university	1.0	
	Did not complete college or university	4.7 (1.4, 16.3)	0.01
**Occupation ISCO classification skill level**	3/4	1.0	
	2	1.5 (0.2, 11.0)	0.67
	1	3.8 (0.6, 22.7)	0.15
	Student	1.1 (0.2, 5.5)	0.90
	Unemployed	6.6 (1.4, 31.7)	0.02

Patients with a preoperative diagnosis of keratoconus had a lower incidence of graft failure compared to each of the other three diagnoses. The incidence also was lower in patients who had completed college or university. When all variables from Table 3 were considered in a multivariable Cox regression model using stepwise variable selection, preoperative diagnosis and education were the only variables selected (p = 0.05 and p = 0.03, respectively).

The distribution of visual acuity before surgery and at postoperative year one is shown in Table 4. Of the eyes that made it to followup one year postoperatively, 85.3% achieved improved, 4.4% achieved the same, and 10.3% achieved worse uncorrected visual acuity (UCVA) compared to before surgery.

**Table 4:** Preoperative and 1-year postoperative visual acuity.

	Preoperative	1 year Postoperative
**Visual acuity**	N (%)	N (%)
≥6/18	0 (0)	24 (32.9)
<6/18-6/36	2 (1.8)	25 (34.3)
<6/36-6/60	9 (8.1)	5 (6.9)
<6/60-3/60	24 (21.6)	2 (2.7)
<3/60	76 (68.5)	17 (23.3)
Total	111*	73

*Preoperative visual acuity was not recorded in 7 patients

Over the study period, 12 patients (10.2%) underwent regraft, 23 (19.5%) underwent at least one rejection episode, 9 (7.6%) underwent traumatic dehiscence, 6 (5.1%) developed infectious keratitis, and 3 (2.5%) developed endophthalmitis. Four eyes (3.4%) were irreversibly lost due to phthisis or need for evisceration or alcohol block.

## Discussion

This paper reports the outcomes of primary penetrating keratoplasty performed in a low-resource setting in rural Kenya. The other report on outcomes of penetrating keratoplasty performed in a sub-Saharan African setting was published by Yorston et al. at Kikuyu Hospital, located in a different region of Kenya [5]. Similar to that report, keratoconus was the leading surgical indication in our population. The preponderance of keratoconus in both of these reports from Kenya is in contrast to a large series published by Dandona et al. on penetrating keratoplasty performed at the L.V. Prasad Eye Institute in Hyderabad, India, where corneal scar was the leading indication at 23.4% of the study population [10].

The overall one-year graft survival rate was 85.8%. This is higher than the one-year survival rate reported by Dandona et al. of 79.6% [10], due to the higher proportion of keratoconus in our study population. Consistent with their study and other studies in both developed and developing countries [5, 10–13], keratoconus in our study had the highest graft survival rate compared to other preoperative diagnoses. The one-year survival rate for keratoconus in our study was 89.9%, compared with 96.4% in Dandona et al., and, although not explicitly reported by Yorston et al., is similar to their reported two-year graft survival rate of 87.4% [5, 10].

Consistent with a published report on keratoconus in Kenya [14], we observed that the age of presentation for keratoconus at our center can be early, with advanced disease sometimes presenting even in the first decade. This may be at least in part due to the association of keratoconus with vernal keratoconjunctivitis [15], which has been shown to occur in high prevalence in children in other African countries [16, 17], and consistent with the authors’ clinical observations at Tenwek Hospital. Although the one-year graft survival rate for those age 14 and younger was lower than that of those older than age 14, there was no statistically significant difference between the groups based on Cox regression modeling, and due to the small numbers of those in the younger group it is not possible to draw any definitive conclusions regarding age and graft survival. Keratoconus in the pediatric population appears to be more aggressive than keratoconus in the adult population [18]. The earlier age of presentation of keratoconus in Kenya poses a difficult societal challenge, given that affected children are becoming visually impaired during the years of primary and secondary education, often precluding them from continuing their education. In this study population as a whole, at 1 year, 85.3% had an improvement in UCVA and 32.9% achieved a UCVA of ≥6/18, which by the World Health Organization standards is considered mild to no visual impairment [19]. This study reports UCVA, as the uncorrected value is most clinically relevant to this patient population where proper spectacle fitting is limited and contact lenses are out of reach of the majority. While corneal transplantation may allow a child to return to school, we remain ambivalent whether this is a sustainable solution to address the suspected high number of children with keratoconus in Kenya. The question remains regarding the longevity of the transplant and the need for repeat transplants at a later date. This highlights the importance of early screening and the establishment of corneal crosslinking services to reduce the number of patients needing corneal transplantation. This also highlights the importance of the establishment of resources for the proper fitting of spectacles and contact lenses, as these may restore functional vision in earlier forms of keratoconus and delay the need for transplantation, as well as improve visual acuity post transplantation.

This paper adds to published knowledge that patient socioeconomic characteristics may be associated with graft failure. In their study population in Hyderabad, India, Dandona et al. found that patients of lower socioeconomic status had a 1.28 times higher risk of graft failure compared with patients of higher socioeconomic status [10]. In a nationwide study using the United Kingdom Transplant Registry, Chua et al. found that the risk of graft failure within 5 years post operation of patients classified as hard-pressed was 1.3 times higher than that of patients classified as wealthy achievers [20]. In this study, we found that compared to patients who completed college or university, the risk of graft failure was 4.7 times higher among patients with less education. Those who were unemployed were 6.6 times more likely to have graft failure compared with those who had higher skilled occupations as classified by the ISCO. In spite of being a rural mission hospital, due to the limitation of corneal transplantation services in Kenya, Tenwek Hospital attracts a wide patient demographic, including a significant number of patients from the capital city Nairobi (16.7% in this study), and those who are more educated than the general Kenyan population. In Kenya where only 5% are educated beyond secondary school [21], 43.0% of our population had either completed college or university. While the notion that those of lower socioeconomic status have a higher chance of graft failure may make intuitive sense, the reasons for this are not as clear and may be related to factors not explored in this study, such as poor patient compliance and delayed treatment due to a greater tolerance of ill health. While most charity and subsidized eye care focuses on the most disenfranchised, the findings in this paper suggest that perhaps, especially in light of the limited supply of corneas, those of lower socioeconomic status in developing countries may not be the optimal target population to receive corneal transplantation. This being said, the establishment of corneal transplantation services in a society may indirectly benefit the most disenfranchised in the long-term. As the provision of corneal transplantation for those of higher socioeconomic status may help grow the eye care infrastructure in low-resource settings, other eye care services, such as spectacles and cataract surgery, may become more readily available for all.

### Limitations

There are several limitations to this study, including the small number of eyes relative to other outcome studies [10, 13, 22]. This is further compounded by the large proportion of the study population (33.9%) that failed to make it to followup one year postoperatively. The relatively small number of eyes in this study accurately reflects how rarely corneal transplantation is performed in Kenya, as Tenwek Hospital, over the study period, was one of the leading providers of corneal transplantation in Kenya. The large loss to followup rate captures the challenging nature of performing corneal transplantation on this particular patient population, as this loss to followup occurred despite every patient providing verbal commitment to followup at Tenwek Hospital for at least one year. The reasons that patients failed to followup are beyond the scope of this report. One can conjecture that patients did not followup if they were doing well and did not find the need. However, another possibility is that they were doing poorly and lost the confidence to return, in which case the rate of adverse outcomes may be under reported. This is likely not the case, as the vast majority of those who were lost to followup expressed satisfaction with the procedure on the telephone questionnaire. This issue of significant loss to followup must be taken into consideration when offering this surgery in this patient population, and further investigation is necessary to determine the reasons for poor followup as well as methods to promote better adherence to followup recommendations.

This study brings attention to the challenges of clinical documentation in a mission hospital setting. Even with the implementation of an electronic health record system, at times essential clinical information, such as the preoperative visual acuity in 7 patients, though certainly performed, was not recorded. This oversight may have been due to lack of attention to detail due to the overwhelming patient load of a mission hospital. Some details relevant to graft survival were either not recorded or not able to be determined. The degree of corneal vascularization preoperatively, which is known to affect the prognosis of the graft, was not recorded as it is in other studies [10, 22, 23]. Also, although it is well accepted that corneal scars due to herpetic causes fare worse prognostically compared to those due to other causes, given that patients in this study had limited if any eye care prior to initial presentation and often presented with advanced forms of disease, this differentiation could not be determined.

## Conclusions

This study highlights several challenges of corneal transplantation in rural Kenya, both in providing the service and also in analyzing the outcomes. Despite these challenges, this paper supports the notion that there may be a select patient population where corneal transplantation has a reasonable chance of success, and this population should be the first to be targeted. The findings of this study suggest that additional studies of larger patient populations with longer followup periods in similar settings may be useful in informing the best practices for appropriate patient selection to maximize successful outcomes of corneal transplantation in low-resource settings.

## Supporting information

S1 File Study data set. (XLSX)
